# Immunotherapies for Anti-N-M-methyl-D-aspartate Receptor Encephalitis: Multicenter Retrospective Pediatric Cohort Study in China

**DOI:** 10.3389/fped.2021.691599

**Published:** 2021-06-29

**Authors:** Shiqi Guang, Jiannan Ma, Xiaotun Ren, Shuizhen Zhou, Jian Yang, Jianzhao Zhang, Xiaoshuang Cao, Linxiu Zhong, Xiao Ding, Xiaosu Wang, Changhong Ren, Weihua Zhang, Linmei Zhang, Min Zhang, Jing Sun, Miriam Kessi, Fei Yin, Jing Peng, Yuwu Jiang

**Affiliations:** ^1^Department of Pediatrics, Xiangya Hospital of Central South University, Hunan Intellectual and Developmental Disabilities Research Center, Changsha, China; ^2^Department of Neurology, Children's Hospital of Chongqing Medical University, National Clinical Research Center for Child Health and Disorders, Ministry of Education Key Laboratory of Child Development and Disorders, Chongqing Key Laboratory of Translational Medical Research in Cognitive Development and Learning and Memory Disorders, Chongqing, China; ^3^Department of Neurology, Beijing Children's Hospital, National Center for Children' Health, Capital Medical University, Beijing, China; ^4^Department of Neurology, Children's Hospital of Fudan University, Shanghai, China; ^5^Children's Hospital Affiliated to the Capital Institute of Pediatrics, Beijing, China; ^6^Division of Pediatric Neurology, Pediatrics Department, Peking University First Hospital, Beijing, China

**Keywords:** N-methyl-D-aspartate receptor, anti-NMDAR encephalitis, autoimmune encephalitis, pediatrics, immunotherapy, prognosis

## Abstract

**Background:** Anti-N-methyl-D-aspartate receptor (NMDAR) encephalitis has been discovered for more than a decade, but the establishment of standardized immunotherapy protocol for pediatric patients still needs more clinical evidence.

**Methods:** A multicenter, retrospective study was conducted on pediatric patients diagnosed with anti-NMDAR encephalitis between November 2011 and December 2018. The clinical records including clinical manifestations, immunotherapy strategies, and outcomes were collected and analyzed.

**Results:** A total of 386 patients were included in our study and the median onset age was 8.00 (IQR 4.83–10.90) years. All patients received first-line immunotherapy and the majority (341, 88.3%) used the standard combination of methylprednisolone pulses (MEP) and intravenous immunoglobulins (IVIG), but 211 patients did not show satisfactory improvement (mRS ≥ 3). Mainly three treatment strategies were applied after first-line immunotherapy: second-line immunotherapy, repetitive first-line immunotherapy, and maintaining oral prednisolone. For patients with mRS ≥ 4 after first-line immunotherapy, the incidence of poor outcome (mRS ≥ 3) in oral prednisolone group was higher than that in other treatment groups (*p* = 0.039). No difference in complete recovery rate (mRS = 0) was found between patients receiving second-line and repetitive first-line immunotherapy, or patients using long-term and short-term prednisolone. Out of 149 patients who received anti-myelin oligodendrocyte glycoprotein antibody (MOG-Ab) test, 27 (18.12%) were positive. Patients with concomitantly positive MOG-Ab showed milder conditions compared to patients with typical anti-NMDAR encephalitis and were more inclined to relapses. We also identified female, MOG-Ab positive, and not receiving second-line and/or repetitive first-line immunotherapy were risk factors for relapses.

**Conclusions:** For patients with mRS ≥ 4 after first-line immunotherapy and patients with concomitantly positive MOG-Ab, second-line immunotherapy is recommended. When second-line immunotherapy is not applicable, repetitive first-line immunotherapy can be considered as an option. Both second-line and repetitive first-line immunotherapy are beneficial to reduce relapse rate. The duration of sequential oral prednisolone can be shortened after fully evaluating patients' conditions.

## Introduction

Anti-N-methyl-D-aspartate receptor (NMDAR) encephalitis was first described in 2007 due to the discovery of specific autoantibodies to NMDA receptors in a series of patients who developed a constellation of neuropsychiatric symptoms, associated with ovarian teratoma (1, 2). A large series study of 577 patients revealed approximately 80% of patients improved or recovered after immunotherapies and tumor removal if it was applicable (3). Compared to adult patients, pediatric patients showed a different clinical symptom profile and lower tumor association rate, and they may have different response to immunotherapy and better outcome (4). Although the frame of immunotherapy is well-defined, in which patients receive first-line immunotherapy and then proceed to second-line immunotherapy if clinical improvement is not satisfactory, the evidence of effectiveness and superiority of any treatment regimen is lacking. Currently, the most widely accepted first-line immunotherapy is high-dose intravenous methylprednisolone pulses (MEP) alone or combined with intravenous immunoglobulins (IVIG); rituximab for second-line immunotherapy is another area of agreement (5, 6). However, our survey on treatment strategies of pediatric neurologists in China revealed a considerable proportion of clinical practitioners would repeat first-line immunotherapy once before considering second-line immunotherapy (7). The survey of Bartolini et al. also showed pediatric neurologists were more likely to repeat first-line immunotherapy compared to adult neurologists and pediatric rheumatologists (5). High dose of oral prednisolone followed by tapering is another major maintenance treatment after first-line immunotherapy, but the dosage and duration are also open to discussion. How these clinical decisions will affect the outcome of patients is still a pending question. Furthermore, co-existing myelin oligodendrocyte glycoprotein antibody (MOG-Ab) in patients diagnosed with anti-NMDAR encephalitis is not rare, and this subpopulation of patients may have different response to treatment according to previous studies (8–10).

Consequently, we performed this nationwide, multicenter study with 386 pediatric patients involved, aiming to investigate the current immunotherapy strategies for pediatric patients with anti-NMDAR encephalitis in China, how different immunotherapies affect the long-term prognosis of patients, and how patients with positive MOG-Ab responded to immunotherapy.

## Materials and Methods

### Study Design

Pediatric patients diagnosed with anti-NMDAR encephalitis between November 2011 and December 2018 from Xiangya Hospital of Central South University, Children's Hospital of Chongqing Medical University, Peking University First Hospital, Beijing Children's Hospital, Children's Hospital of Fudan University, and Capital Institute of Pediatrics were enrolled in our study. Requirement for informed consent was waived by the Ethics Committee of each institution due to the retrospective nature of the study.

The inclusion criteria were: (1) meeting the diagnostic criteria for anti-NMDAR encephalitis (11, 12), (2) age under 18 years old, and (3) follow-up duration > 12 months (unless the patient died from anti-NMDAR encephalitis). The exclusion criteria included: (1) lacking key clinical data, (2) being diagnosed with autoimmune diseases such as rheumatoid arthritis and lupus, or other brain disorders. Medical records including demographics, clinical characteristics, CSF examinations, brain MRI, electroencephalography (EEG), systemic screening for potential tumors, immunotherapies and relapses, as well as long-term outcome were reviewed. The follow-up was conducted through outpatient visits or telephone interviews. Treatment response and outcome analysis were assessed with mRS during follow-up. Patients were considered to have a good outcome if mRS ≤ 2 at the last follow-up and a poor outcome if mRS ≥ 3. Complete recovery was defined as mRS = 0. Relapse was defined as any new onset neurologic, psychiatric symptoms, or the worsening of the pre-existing symptoms after stabilization or improvement for 2 months, which could not be explained by other causes.

### Statistical Analyses

Statistical analyses were performed using IBM SPSS Statics 26.0 (IBM Corp, Armonk, NY, USA). Figures were generated with GraphPad Prism 8.0.2 (GraphPad Software, San Diego, CA, USA) and R package “ggplot2 (3.3.3).” Quantitative data of demographic features and clinical records were presented as median with interquartile range (IQR). Categorical variables of patients with good and poor outcome were compared using Chi-squared test, continuity correction or Fisher's exact test where applicable; continuous variables were analyzed with Mann-Whitney U-test. Predictor variables with *p* ≤ 0.1 in univariate analysis were included for multivariate logistic regression analysis.

## Results

### Demographic and Clinical Manifestations

A total of 386 patients were enrolled, and 224 (58.0%) were females. The median age of symptom onset was 8.00 (IQR 4.83–10.90) years. Four patients (1.0%) were found to have an associated tumor, with two having teratoma, one having optic glioma, and one having Ewing's sarcoma. All patients had mRS evaluated at symptom onset, with 324 (83.9%) patients having mRS ≥ 3. CSF examinations showed all patients had positive anti-NMDAR antibodies, and 183 (47.4%) patients showed other CSF abnormalities, such as mild to moderate pleocytosis (white cell count > 5 mm^3^) and increased level of proteins (CSF proteins > 450 mg/L). Abnormal signals in brain MRI were shown in 190 (49.2%) patients. The most frequently affected locus was cerebral cortex (54.2%), and other involved areas were basal ganglia, thalamus, hippocampus, white matter, and cerebellum. Abnormal EEG activities were identified in 306 (79.3%) patients, including epileptic discharges, diffuse slow activity, and focal slow activity.

Myelin oligodendrocyte glycoprotein antibody test was conducted in 149 patients, and 27 (18.12%) were identified to have concomitant positive MOG-Ab in CSF. Compared to typical anti-NDMAR encephalitis, we found patients with positive MOG-Ab had milder conditions, such as lower incidence of movement disorder (*p* = 0.003), seizures (*p* = 0.047), and lower PICU admission rate (*p* = 0.027), as well as better response to first-line immunotherapy (*p* = 0.036) (Table 1). Univariate analysis also showed patients with positive MOG-Ab had higher relapse rate (*p* = 0.009).

**Table 1:** Comparison of clinical characteristics between patients with concomitant positive MOG-Ab and patients with typical anti-NMDAR encephalitis.

	**Patients with concomitant positive MOG-Ab (*N* = 27), *n* (%)**	**Patients with typical anti-NMDAR encephalitis (*N* = 119), *n* (%)**	**Univariate analysis**		**Multivariate analysis**	
			***z*-value/χ^**2**^**	***p-*value**	**OR (95% CI)**	***p-*value**
Female	16 (59.26)	69 (57.98)	0.015	0.903^a^	Not selected	
Tumor	0 (0)	2 (1.68)	-	1.000^b^		
Age at symptom onset, median (IQR)	8.25 (5.44–9.86)	8.00 (4.95–11.75)	−0.676	0.499^c^		
Prodromal symptoms	17 (62.96)	55 (46.22)	2.469	0.116^a^		
Having fever within 3 weeks before symptom onset	12 (44.44)	43 (36.13)	0.647	0.421^a^		
Behavioral change	21 (77.78)	105 (88.24)	1.247	0.264^d^		
Movement disorder	16 (59.26)	103 (86.55)	9.142	0.002^d^	5.770 (1.788–18.620)	0.003
Speech disorder	19 (70.37)	83 (69.75)	0.004	0.949^a^		
Seizures	16 (59.26)	92 (77.31)	3.725	0.054^a^	2.814 (1.013–7.815)	0.047
Decreased level of consciousness	8 (29.63)	63 (52.94)	4.787	0.029^a^	0.754 (0.247–2.296)	0.619
Autonomic dysfunction	0 (0)	15 (12.61)	2.549	0.110^d^	Not selected	
Abnormal brain MRI	13 (48.15)	59 (49.58)	0.168	0.682^a^		
Abnormal EEG	23 (85.19)	96 (80.67)	0.073	0.787^d^		
Abnormal CSF examinations*	18 (66.67)	68 (57.14)	0.825	0.364^a^		
mRS at symptom onset [median, (IQR)]	3 (2–4)	4 (3, 4)	−1.435	0.151^c^		
mRS after first-line immunotherapy [median, (IQR)]	2 (0–3)	3 (2–4)	−3.129	0.002^c^	0.680 (0.474–0.975)	0.036
Days of interval between symptom onset and treatments [median, (IQR)]	26.00 (14.50–40.50)	21.00 (12.00–34.00)	0.661	0.509^c^	Not selected	
Requires of PICU admission	1 (3.70)	35 (29.41)	7.829	0.005^a^	13.381 (1.353–132.336)	0.027
Relapse	7 (25.93)	8 (6.72)	6.743	0.009^d^	0.647 (0.168–2.492)	0.527
Good outcome	26 (96.30)	109	0.000	0.992^d^	Not selected	
Follow-up days [median, (IQR)]	1,297.00 (808.50–2,025.00)	1,262.00 (922.00–2,025.00)	0.335	0.737^c^		

a *Pearson's χ^2^-test*.

b *Fisher's exact test*.

c *Mann-Whitney U-test*.

d *Chi-squared test with continuity correction*.

### Immunotherapies

**Figure 1:**
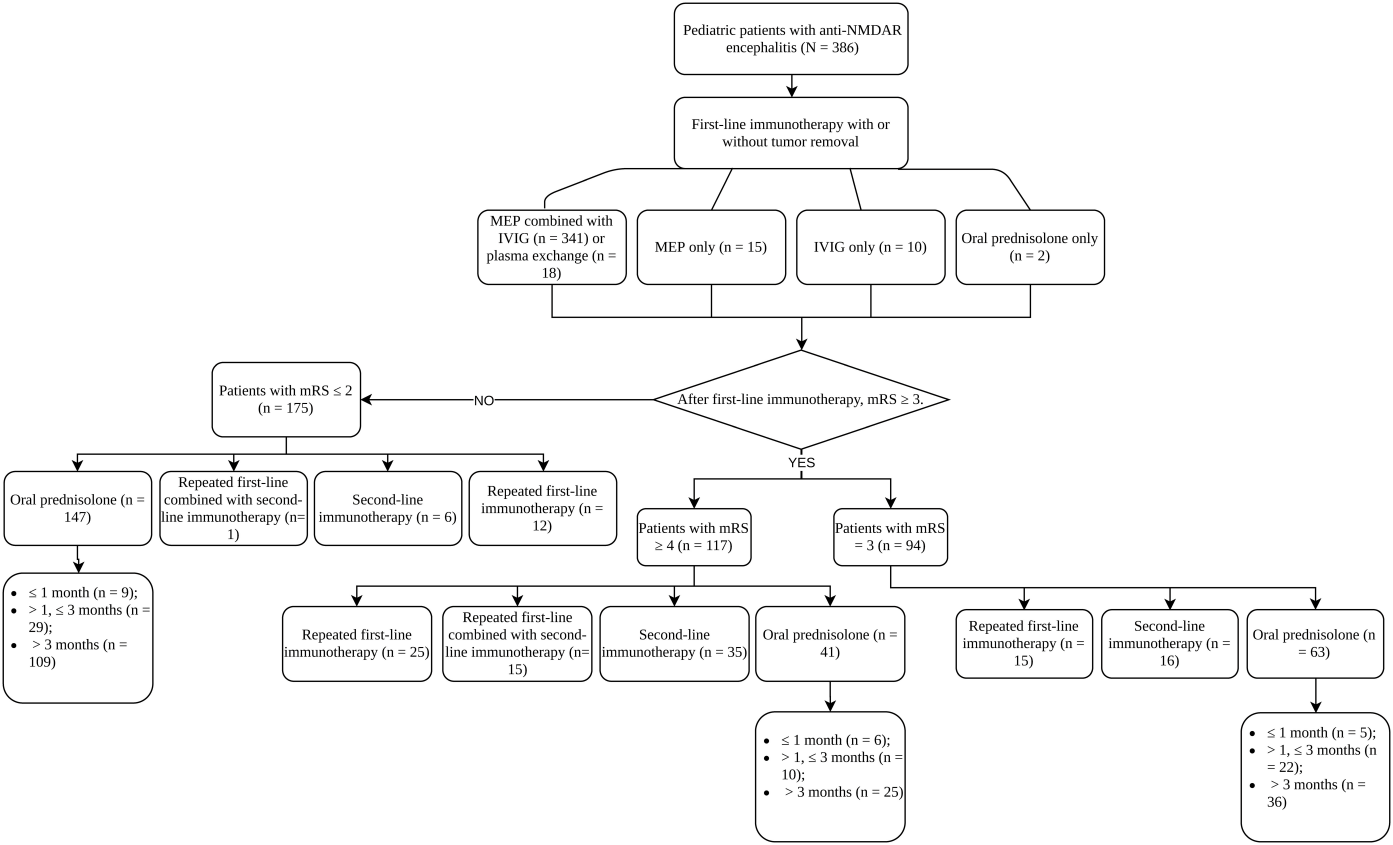
Treatment strategies of patients with anti-NDMAR encephalitis.

**Figure 2:**
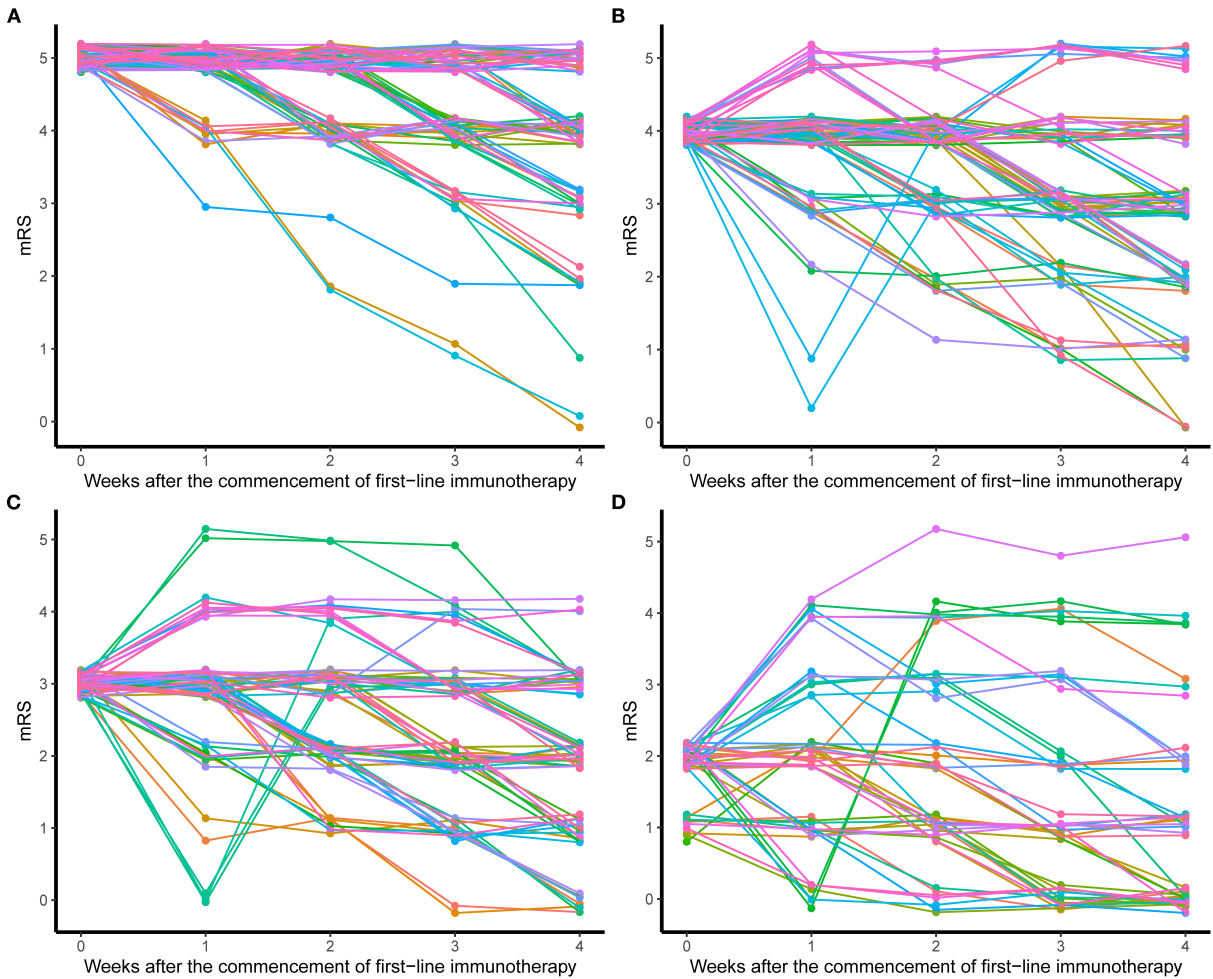
mRS trajectories of patients after the commencement of first-line immunotherapy. **(A)** Patients with initial mRS = 5. **(B)** Patients with initial mRS = 4. **(C)** Patients with initial mRS = 3. **(D)** Patients with initial mRS ≤ 2.

The treatment processes of all patients were summarized in Figure 1. The majority of patients (341, 88.3%) used the combination of MEP and IVIG. Eighteen patients (4.7%) additionally received plasma exchange (PLEX) on the basis of MEP. Fifteen (3.9%) and 10 (2.6%) patients received MEP and IVIG only, respectively. For 374 patients who used MEP treatment, 363 (97.06%) had the dosage higher than 10 mg/m^2^/day. The duration of MEP was limited within 5 days in 265 (70.86%) patients. After MEP was completed, except for one patient deceased within 1 week of first-line immunotherapy, and patients who received IVIG only, 375 (96.6%) patients sequentially received high-dose oral prednisolone (≥ 2 mg/kg/d or 60 mg/d). Almost half of the patients (176, 46.9%) limited the use of high-dose prednisolone to 2 weeks, and 75 (20%) kept the high dosage for 2 weeks to 1 month. During this period, treatment response of first-line immunotherapy was evaluated once per week starting from the first week to the fourth after the commencement of first-line immunotherapy and 264 (68.8%) patients had mRS available for all time points. mRS trajectories (Figure 2) of these patients revealed that the conditions of patients might worsen within the first week, and some patients, although only in a small number, who had decent response in the first week could have rebound in the following week. Good response to first-line immunotherapy (mRS ≤ 2) was achieved in 175 patients, whereas 211 (53.9%) patients did not show satisfactory improvement (mRS ≥ 3).

On the basis of oral prednisolone, mainly three treatment strategies were applied after first-line immunotherapy: adding second-line immunotherapy, repeating first-line immunotherapy, and maintaining oral prednisolone only. For patients who repeated first-line immunotherapy, 48.1% received the standard combination of MEP and IVIG, and 44.2% used IVIG only. The profile of repetitive first-line immunotherapy was quite different from the first time, indicating when choosing repetitive first-line immunotherapy, clinical practitioners tended to use the milder regimen of monotherapy. Fifty-seven out of 73 (78.1%) patients started second-line immunotherapy within 4 weeks, including 35 starting within 2 weeks. Interval between first- and second-line immunotherapy longer than 4 weeks usually resulted from the use of repetitive first-line immunotherapy.

We grouped patients according to the response to first-line immunotherapy and summarized their treatments. In the group of patients with mRS ≥ 4 (*n* = 117), 25 (21.4%) received repetitive first-line immunotherapy, and 35 (29.9%) patients received second-line immunotherapy directly. However, 15 (12.8%) patients received both because they did not show clinical improvement after repeating first-line immunotherapy. For the patients with mRS = 3 (*n* = 94), 15 (16.0%) patients received repetitive first-line immunotherapy, and 16 (17.0%) patients used second-line immunotherapy. As for the group of patients with mRS ≤ 2 (*n* = 175), the proportions of patients that received repetitive first-line immunotherapy (6.9%) and second-line immunotherapy (3.4%) were lower than that of the other two groups. Only one patient had to go through both treatments. Forty-one (35.0%), 63 (67.0%), and 147 (84.0%) patients maintained oral prednisolone in the groups of mRS ≥ 4, mRS = 3, and mRS ≤ 2, respectively. Among these patients (*n* =251), 170 (67.7%) used long-term treatment course (> 3 months), whereas 61 (24.3%) used prednisolone for 1–3 months, and 20 (8.0%) tapered and stopped prednisolone within 1 month. Eighteen patients (4.7%) received long-term immunosuppressive treatments in our series, 15 using mycophenolate mofetil, 2 using azathioprine, and 1 patient receiving both, and 11 of them maintained chronic immunotherapy for more than 1 year.

### Outcome

The median duration of follow-up in our study reached to 38.6 (IQR 24.73–49.53) months. Five (1.3%) patients were lost to follow-up. We were able to assess the final mRS in 381 (98.7%) patients, and 360 (94.5%) achieved good outcome (mRS ≤ 2) including 270 patients who had complete recovery (mRS = 0). By the last follow-up, 21 (5.4%) patients had poor outcome (mRS ≥ 3) and 6 (1.6%) of them died of this disease. The comparison of clinical characteristics of patients with good and poor outcome was shown in Table 2. The patients with poor outcome were more likely to be younger, have prodromal symptoms, especially fever within 3 weeks before onset, and have higher mRS at symptom onset. During the disease course, the patients with poor outcome had higher rate of decreased level of consciousness, abnormal brain MRI, and unsatisfactory response to first-line immunotherapy. Pediatric intensive care unit (PICU) admission was also more frequently required in the patients with poor outcome. Patients with good outcome had longer follow-up time; however, the deceased patients we included biased long-term evaluation of living patients, because all these patients died at the relatively early course of disease [median 52 (IQR 30–100) days, Supplementary Figure 1]. If excluding the deceased patients, the follow-up time between patients with good and poor outcome was not different statistically. Younger age and unsatisfactory response to first-line immunotherapy were independent predictors to poor outcome after performing multivariate logistic regression.

**Table 2:** Comparison of clinical characteristics of patients with good and poor outcome.

	**Good outcome (mRS ≤ 2) (*N* = 360), *n* (%)**	**Poor outcome (mRS ≥ 3) (*N* = 21), *n* (%)**	**Univariate analysis**		**Multivariate analysis**	
			**z-value/χ^**2**^**	***p-*value**	**OR (95% CI)**	***p-*value**
Female	210 (58.3)	12 (57.1)	0.012	0.914^a^	Not selected	
Tumor	4 (1.1)	0 (0)	–	1.000^b^		
Age at symptom onset, median (IQR)	8.13 (5.18–11)	3.9 (1.7–8)	3.420	0.001^c^	1.207 (1.053–1.382)	0.007
Prodromal symptoms	186 (51.7)	18 (85.7)	9.247	0.002^a^	2.169 (0.198–23.808)	0.526
Having fever within 3 weeks before symptom onset	129 (35.8)	17 (81.0)	17.090	0.000^a^	2.133 (0.233–19.520)	0.503
Behavioral change	305 (84.7)	18 (85.7)	0.000	1.000^d^	Not selected	
Movement disorder	281 (78.1)	17 (81.0)	0.002	0.968^d^		
Speech disorder	263 (73.1)	17 (81.0)	0.635	0.425^a^		
Seizures	262 (72.8)	17 (81.0)	0.676	0.411^a^		
Decreased level of consciousness	184 (51.1)	17 (81.0)	7.089	0.008^a^	0.916 (0.233–19.520)	0.901
Autonomic dysfunction	35 (9.7)	1 (3.8)	0.138	0.710^d^	Not selected	
Abnormal brain MRI	168 (46.7)	18 (85.7)	11.774	0.001^a^	2.425 (0.603–9.750)	0.212
Abnormal EEG	283 (78.6)	18 (85.7)	0.251	0.616^d^	Not selected	
Abnormal CSF examinations^*^	168 (46.7)	10 (47.6)	0.007	0.932^a^		
mRS at symptom onset [median, (IQR)]	3 (3,4)	4 (4,5)	−3.851	0.000^c^	0.934 (0.467–1.868)	0.846
mRS after first-line immunotherapy [median, (IQR)]	3 (1–4)	4 (4,5)	−5.182	0.000^c^	0.423 (0.236–0.759)	0.004
Days of interval between symptom onset and treatments [median, (IQR)]	21 (12.5–34)	23 (11–34)	−0.305	0.760^c^	Not selected	
Requires of PICU admission	49 (13.6)	8 (38.1)	7.524	0.006^d^	2.381 (0.755–7.507)	0.139
Follow-up days [median, (IQR)]	1,176.5 (782.5–1,516.5)	779 (164–1,318)	−2.513	0.012^c^	Not selected	
Follow-up days [median, (IQR)]	1,176.5 (782.5–1,516.5)	1,148 (760.5–1,448.5)^&^	−0.372	0.710^c^		

a *Pearson's χ^2^-test*.

b *Fisher's exact test*.

c *Mann-Whitney U-test*.

d *Chi-squared test with continuity correction*.

* *CSF white cell count >5/mm^3^ and/or CSF protein levels >450 mg/L.*

& *Six deceased patients were ruled out from analysis*.

We identified unsatisfactory response to first-line immunotherapy as the independent predictor to poor outcome (mRS ≥ 3) (Table 2). Then we changed outcome event to complete recovery (mRS = 0) and analyzed how mRS at onset and after first-line immunotherapy affected the outcome within each treatment group (Supplementary Table 1). In the groups of second-line immunotherapy (*p* = 0.044) and oral prednisolone (*p* = 0.001), the complete recovery rate of patients with mRS ≥ 4 after first-line immunotherapy was lower than that of patients with mRS ≤ 3. Therefore, we grouped patients based on their response to first-line immunotherapy and analyzed the effects of different treatment strategies on patient outcome. For patients with mRS ≥ 4 after first-line immunotherapy, the incidence of poor outcome in oral prednisolone group was higher than other treatment groups (Table 3, *p* = 0.039). In the groups of patients with mRS = 3 and ≤ 2, there was no significant difference in complete recovery rate between patients from oral prednisolone group and other treatment groups (Table 3). Patients using long-term (> 3 months) and short-term (≤3 months) prednisolone showed no difference in complete recovery rate (Table 4). Moreover, patients receiving second-line, repetitive first-line immunotherapy had no difference in complete recovery rate (Table 5). It is worth noting that in the group of mRS ≥ 4, 15 of 40 patients who repeated first-line immunotherapy had to receive second-line immunotherapy due to poor improvement, but this subset of patients showed no difference in complete recovery rate compared to patients from other treatment groups.

**Table 3:** Comparison of effects of oral prednisolone and other treatments on patient outcome.

	**Good outcome (mRS ≤ 2)**	**Poor outcome (mRS ≥ 3)**	***χ2***	***p*-value**
mRS ≥ 4 after first-line immunotherapy			4.259	*p* = 0.039
Oral prednisolone	30	9		
Second-line and/or repetitive first-line immunotherapy	70	7		
mRS = 3 after first-line immunotherapy			0.60	*p* = 0.333
Oral prednisolone	58	2		
Second-line and/or repetitive first-line immunotherapy	28	3		
	**Complete recovery (mRS** **=** **0)**	**Incomplete recovery (mRS** **≥** **1)**		
mRS ≤ 2 after first-line immunotherapy			0.582	*p* = 0.467
Oral prednisolone	115	24		
Second-line and/or repetitive first-line immunotherapy	27	8		

**Table 4:** Comparison of effects of long-term and short-term prednisolone on patient outcome.

	**Complete recovery (mRS = 0)**	**Incomplete recovery (mRS ≥1)**	***χ2***	***p*-value**
mRS ≥ 3 after first-line immunotherapy			0.314	*p* = 0.575
≤3 months	33	9		
>3 months	42	15		
mRS ≤ 2 after first-line immunotherapy			0.012	*p* = 0.912
≤3 months	30	6		
>3 months	85	18		

**Table 5:** Comparison of effects of second-line and repetitive first-line immunotherapy on patient outcome.

	**Complete recovery (mRS = 0)**	**Incomplete recovery (mRS ≥ 1)**	***χ2***	***p*-value**
mRS ≥ 4 after first-line immunotherapy			0.044	*p =* 0.978
Second-line immunotherapy	16	18		
Repetitive first-line immunotherapy	12	15		
Both	7	8		
mRS =3 after first-line immunotherapy			0.814	*p* = 0.367
Second-line immunotherapy	9	4		
Repetitive first-line immunotherapy	9	8		

During the follow-up, 27 (7%) patients experienced relapses, of which 21 (77.78%) were female patients. Twenty-five patients had their first relapse within 1 year of disease onset. Two patients had multiple relapses (2–3 episodes). All patients had equal or lower mRS compared to their first-time onset. Female, concomitant positive anti-MOG antibody, and not receiving second-line or repetitive first-line immunotherapy were independent risk factors for relapse (Table 6). The course of oral prednisolone was not related to relapse. After relapsing, four patients received second-line immunotherapy, eight reinitiated first-line immunotherapy, and three used both treatments. The remaining 12 patients took oral prednisolone without any further treatment. At the last follow-up, the outcome between patients with and without relapses was not different.

**Table 6:** Comparison of clinical characteristics of relapsed and non-relapsed patients.

	**Relapse patients (*N* = 27), *n* (%)**	Non-relapse patients (*N* = 348)^*^, *n* (%)	**Univariate analysis**		**Multivariate analysis**	
			**z-value/χ^2^**	***p-*value**	**OR (95% CI)**	***p-*value**
Female	21	197	4.613	0.032^a^	2.732 (1.010–7.391)	0.048
Tumor	0	4	–	1.000^b^	Not selected	
Age at symptom onset, median (IQR)	9.80 (8.25–11.00)	8.00 (4.73–10.85)	2.371	0.018^c^	1.113 (0.983–1.259)	0.091
Anti-MOG antibody positive	7	20	12.399	0.000^d^	0.146 (0.047–0.460)	0.001
mRS at symptom onset [median, (IQR)]	3 (2.50–4)	4 (3–4.5)	−1.074	0.283^c^	Not selected	
mRS after first-line immunotherapy [median, (IQR)]	2 (0.5–3)	3 (2–4)	−2.908	0.004^c^	0.878 (0.638–1.208)	0.424
Days of interval between symptom onset and treatments [median, (IQR)]	18 (8.5–25.5)	22 (13–34)	−1.848	0.065^c^	0.966 (0.936–0.997)	0.033
Requires of PICU admission	1	49	1.523	0.217^d^	Not selected	
Second-line and/or repetitive first-line immunotherapy	2	124	8.947	0.003^a^	5.221 (1.070–25.490)	0.041
Course of oral prednisolone (days)	135 (94–229)	114 (70–234)	0.781	0.435^c^	Not selected	
Good outcome	25	335	0.183	0.669^d^		
Follow-up days [median, (IQR)]	1,297 (1,017.5–1,455)	1,167.5 (747.5–1,516.5)	0.714	0.475^c^		

a *Pearson's χ^2^-test*.

b *Fisher's exact test*.

c *Mann-Whitney U-test*.

d *Chi-squared test with continuity correction*.

* *Six deceased patients and five patients lost to follow-up were excluded*.

## Discussion

Pediatric patients accounted for approximately 40% of reported cases of anti-NMDAR encephalitis (3, 13). To our knowledge, multicenter studies of immunotherapies of pediatric anti-NDMAR encephalitis across multiple areas in China are rare. At symptom onset, the percentage of patients with mRS ≥ 3 was 83.9%, which was decreased to 54.7% after first-line immunotherapy. Second-line immunotherapy is recommended to administrate to patients who do not have good response to first-line immunotherapy. However, using mRS to assess treatment response can only reflect the conditions of patients at specific time points but fails to show the tendency of the disease. Tracking mRS trajectory is helpful to predict the change of disease. Our study showed a considerable proportion of patients did not reach the extreme stage of disease when hospitalized, and their conditions worsened in the first week after treatment. Moreover, some patients could have rebound, and even they had fast response to first-line immunotherapy. Therefore, it is more conservative to evaluate treatment effect after 3–4 weeks of first-line immunotherapy. In reality, prediction to the progression of disease from practitioners' perspective, as well as many other factors, such as costs, hospitalization requirements, and concerns about side effects, have to be taken into consideration when applying second-line immunotherapy. In mRS ≥ 4 group, patients taking oral prednisolone showed unfavorable outcome compared to patients who received second-line and/or repetitive first-line immunotherapy. Therefore, for patients with mRS ≥ 4 after first-line immunotherapy, aggressive treatments are recommended and use of oral prednisolone without any other treatments should be avoided. Patients using second-line and repetitive first-line immunotherapy did not show significant difference in complete recovery rate and 37.5% of patients who repeated first-line immunotherapy still needed second-line immunotherapy due to unsatisfactory improvement, but this subset of patients showed no difference in complete recovery rate compared to patients from other treatment groups, suggesting repetitive first-line immunotherapy can be considered when second-line immunotherapy is not applicable due to severe adverse effects or high costs, and delaying second-line immunotherapy in patients with repetitive first-line immunotherapy did not affect outcome.

The patients with co-existing MOG-Ab tended to have milder conditions than ones with typical anti-NMDAR encephalitis, in agreement with previous studies (8, 10). Patients with MOG-Ab showed higher relapse rate [Table 1, 25.93 vs. 5.21%, OR = 0.216 (95% CI 0.075–0.623), *p* = 0.005]. Comparing relapsed and non-relapsed patients, we identified female, concomitant positive anti-MOG antibody, and not receiving second-line and/or repetitive first-line immunotherapy were independent risk factors for relapsing (Table 6). Based on our findings, patients with concomitantly positive MOG-Ab and anti-NMDAR antibody are a common subpopulation of autoimmune encephalitis, who can present milder disease conditions but subject to relapses. Second-line or repetitive first-line immunotherapy should be taken into consideration when MOG-Ab is concomitantly positive with anti-NMDAR antibody, even with patients who may have mild conditions and good response to first-line treatment.

More than half of patients who kept on oral prednisolone in our series used a long-term regimen (>3 months). However, long-term use of corticosteroid can cause multiple adverse effects, such as cushingoid features (redistribution of body fat with truncal obesity, buffalo hump, and moon face), osteoporosis, infections, growth impairments, and so on. We found no difference in complete recovery rate between patients using short-term (≤ 3 months) and long-term (>3 months) regimens, and course of oral prednisolone was not related to relapse. Therefore, shortening the duration of prednisolone use is worth considering after fully evaluating patients' conditions.

However, there are limitations to our study. First, selection bias may exist because all hospitals involved in our study are major tertiary hospitals that usually accept more patients with critical conditions than hospitals in a rural area. Some patients were first hospitalized at local hospitals but not tertiary medical centers, thus lacking virological evidence, which made it very challenging to trace the diagnosis of preceding viral encephalitis, although we know it may be related to patient prognosis. Second, evaluation of outcomes was conducted at different time points after treatment across patients. We are aware that recovery from this disease can be slow and take 18 months or longer, and many patients can recover further as follow-up duration is extended. However, we have 329 (85.23%) patients whose follow-up duration was longer than 18 months. If excluding deceased patients for long-term outcome analysis, the follow-up duration was not different between patients with good and poor outcome in our study. Third, the outcome evaluated via mRS is dichotomous, focusing on the locomotor performance. However, multiple studies described pediatric patients could show some neuropsychological sequela, such as cognitive and social functioning deficits, even up until adolescence, causing learning issues (14–16). Thus, prospective longitudinal studies will be required to have better control of these confounding factors to pursue better interventions for this disease.

## Conclusions

In conclusion, for patients with mRS ≥ 4 after first-line immunotherapy and patients with concomitant positive MOG-Ab, we recommend more aggressive treatments like second-line immunotherapy. When second-line immunotherapy is not applicable due to severe adverse effects or other reasons, repetitive first-line immunotherapy can be considered as an option. Both second-line and repetitive first-line immunotherapy are beneficial to reduce relapse rate. The treatment course of sequential oral prednisolone, as a routine maintenance treatment, can be shortened after fully evaluating patients' conditions.

## Data Availability Statement

The raw data supporting the conclusions of this article will be made available by the authors, without undue reservation.

## Ethics Statement

The studies involving human participants were reviewed and approved by Institutional Ethics Committee of Xiangya Hospital of Central South University, Ethical Committee of Beijing Children's Hospital, Ethics Committee of Children's Hospital of Fudan University, Ethics committee of the Children's Hospital of Chongqing Medical University, Ethics Committee of the Peking University First Hospital and Ethics Committee of Capital Institute of Pediatrics. Written informed consent from the participants' legal guardian/next of kin was not required to participate in this study in accordance with the national legislation and the institutional requirements.

## Author Contributions

SG analyzed and interpreted the data, drafted and revised the manuscript for intellectual content. JM, XR, SZ, and JY played the major roles in the acquisition and summarization of data in local hospitals. XC played the major role in the management and summarization of data from all hospitals. LZho analyzed the data and revised the manuscript for intellectual content. JZ, XD, XW, CR, WZ, LZha, MZ, and JS collected clinical records from Electronic Medical Record Systems of local hospitals. MK helped with language polishing. FY revised the manuscript for intellectual content. JP and YJ design and conceptualized study and revised the manuscript for intellectual content. All authors have read and approved the final manuscript.

## Conflict of Interest

The authors declare that the research was conducted in the absence of any commercial or financial relationships that could be construed as a potential conflict of interest.
