# Supplementary material for: Immunotherapies for Anti-N-M-methyl-D-aspartate Receptor Encephalitis: Multicenter Retrospective Pediatric Cohort Study in China
Source: Front Pediatr. 2021 Jun 29;9:691599. doi: 10.3389/fped.2021.691599 (PMC8276978; doi:10.3389/fped.2021.691599)
Supplement: Supplementary file 1 [file Data_Sheet_1.pdf]

Supplementary Table 1. Relationships between mRS at symptom onset or after first-line immunotherapy and patient outcome within each treatment group.

	Complete recovery (mRS = 0)	Incomplete recovery (mRS ≥ 1)	*χ2*	*p* value
Second-line immunotherapy group			1.394	$p = 0.238$
mRS at symptom onset				
mRS ≤ 3	10	4		
mRS ≥ 4	23	20		
mRS after first-line immunotherapy			4.059	$p = 0.044$
mRS ≤ 3	17	6		
mRS ≥ 4	16	18		
Repetitive first-line immunotherapy group				
mRS at symptom onset			0.215	$p = 0.643$
mRS ≤ 3	8	6		
mRS ≥ 4	21	21		
mRS after first-line immunotherapy			1.125	$p = 0.289$
mRS ≤ 3	17	12		
mRS ≥ 4	12	15		
Oral prednisolone group			0.561	$p = 0.756$
mRS at symptom onset				
mRS ≤ 2	43	9		
mRS = 3	74	18		
mRS ≥ 4	73	21		
mRS after first-line immunotherapy			13.006	$p = 0.001$
mRS ≤ 2	115	24		
mRS = 3	52	8		
mRS ≥ 4	23	16		

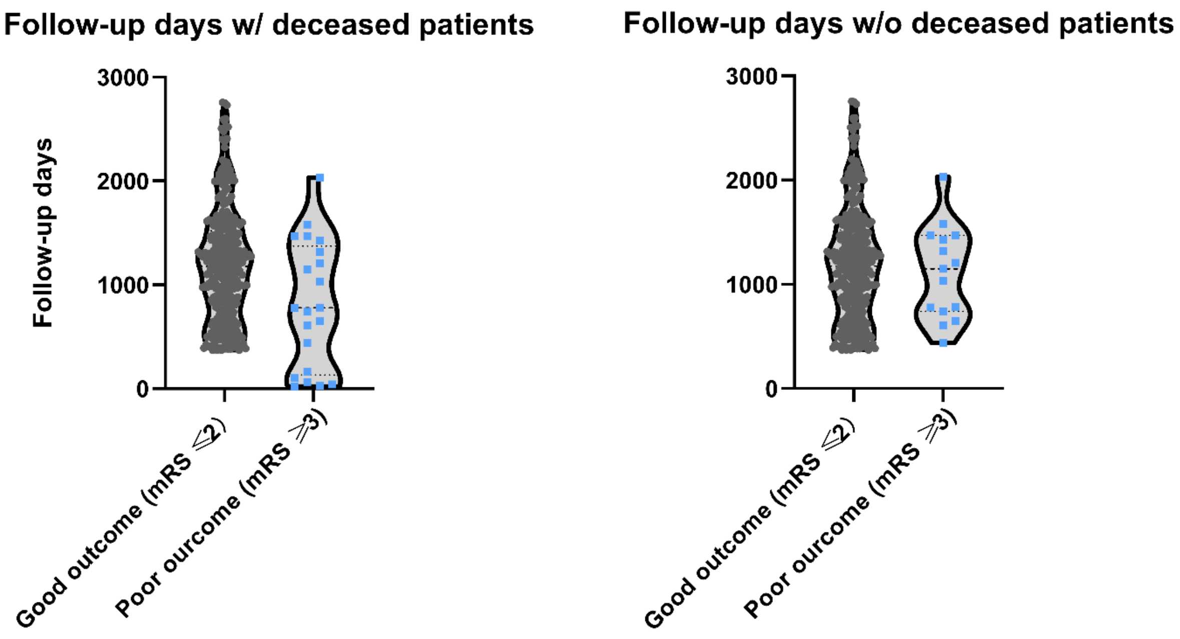

Supplementary Figure 1. Violin plot of distribution of follow-up duration of patients.
